# DNA Methylation-Independent Reversion of Gemcitabine Resistance by Hydralazine in Cervical Cancer Cells

**DOI:** 10.1371/journal.pone.0029181

**Published:** 2012-03-12

**Authors:** Myrna Candelaria, Erick de la Cruz-Hernandez, Lucia Taja-Chayeb, Enrique Perez-Cardenas, Catalina Trejo-Becerril, Aurora Gonzalez-Fierro, Alma Chavez-Blanco, Ernesto Soto-Reyes, Guadalupe Dominguez, Jaenai E. Trujillo, Jose Diaz-Chavez, Alfonso Duenas-Gonzalez

**Affiliations:** 1 Division of Clinical Research, Instituto Nacional de Cancerologia, Mexico City, Mexico; 2 Division of Basic Research, Instituto Nacional de Cancerologia, Mexico City, Mexico; 3 Unit of Biomedical Research in Cancer. Instituto Nacional de Cancerologia/Instituto de Investigaciones Biomedicas UNAM, Mexico City, Mexico; University of South Florida College of Medicine, United States of America

## Abstract

**Background:**

Down regulation of genes coding for nucleoside transporters and drug metabolism responsible for uptake and metabolic activation of the nucleoside gemcitabine is related with acquired tumor resistance against this agent. Hydralazine has been shown to reverse doxorubicin resistance in a model of breast cancer. Here we wanted to investigate whether epigenetic mechanisms are responsible for acquiring resistance to gemcitabine and if hydralazine could restore gemcitabine sensitivity in cervical cancer cells.

**Methodology/Principal Findings:**

The cervical cancer cell line CaLo cell line was cultured in the presence of increasing concentrations of gemcitabine. Down-regulation of *hENT1* & *dCK* genes was observed in the resistant cells (CaLoGR) which was not associated with promoter methylation. Treatment with hydralazine reversed gemcitabine resistance and led to *hENT1* and *dCK* gene reactivation in a DNA promoter methylation-independent manner. No changes in HDAC total activity nor in H3 and H4 acetylation at these promoters were observed. ChIP analysis showed H3K9m2 at *hENT1* and *dCK* gene promoters which correlated with hyper-expression of G9A histone methyltransferase at RNA and protein level in the resistant cells. Hydralazine inhibited G9A methyltransferase activity in vitro and depletion of the G9A gene by iRNA restored gemcitabine sensitivity.

**Conclusions/Significance:**

Our results demonstrate that acquired gemcitabine resistance is associated with DNA promoter methylation-independent *hENT1* and *dCK* gene down-regulation and hyper-expression of G9A methyltransferase. Hydralazine reverts gemcitabine resistance in cervical cancer cells via inhibition of G9A histone methyltransferase.

## Introduction

Gemcitabine (2′,2′-difluoro 2′deoxycytidine, dFdC) is an analog of cytosine arabinoside that possesses distinctive pharmacological properties and wide antitumor-spectrum activity. Gemcitabine has significant clinical activity against a number of malignancies including pancreatic, lung, bladder, breast, ovarian and head and neck [1], [2]. In cervical cancer gemcitabine plus cisplatin and radiation improves survival outcomes compared with cisplatin radiation in advanced disease and when used with cisplatin is as effective as other cisplatin doublets against metastatic cervical cancer [3].

Gemcitabine's pharmacological characteristics are unique in that two main classes of genes are essential for its antitumor effects and resistance: membrane transporter protein-coding genes, whose products are responsible for drug intracellular uptake, and drug metabolism-coding genes, which catalyze its activation and inactivation [4]. Thus, the expression of these genes is key for tumor response and resistance to gemcitabine. Most intracellular uptake of gemcitabine is mediated by hENT1 (human Equilibrative Nucleoside Transporter 1). Sensitivity to nucleoside analogs including gemcitabine *in vitro* and in the clinical setting has been shown to correlate with expression of this transporter whereas hENT1-deficient cells are highly resistant to this nucleoside [5]–[8]. Patients with pancreatic and lung cancer expressing hENT1 have higher response rates and longer median survival after gemcitabine than subjects with low or absent hENT1 [9], [10]. Regarding gemcitabine metabolism genes, *dCK* expression has been associated with gemcitabine sensitivity. Cell lines selected for resistance to nucleoside analogs have shown mutational inactivation of *dCK* and *dCK* transfection results in resensitization of cells to Ara-C and gemcitabine [11]–[12]. In cancer patients a lower expression of dCK is associated with shorter overall survival [13], [14]. On the other hand, diphosphorylated gemcitabine is an inhibitor of ribonucleotide reductase, a heterotetrameric enzyme composed of two homodimers (RRM1 and RMM2) which is key in the synthesis of intracellular deoxynucleotide triphosphate [15]. Over expression of RRM1 and RRM2 has been associated with gemcitabine resistance in cancer cell lines and NSCLC [16], [17]. Cytidine deaminase (CDA) catalyses the deamination of cytidine, dexoycytidine, and their analogs such as gemcitabine however, its role mediating gemcitabine resistance is controversial [18]. These data clearly suggest that a diminished or lack of expression of dCK and hENT1 are crucial for gemcitabine resistance, however, the mechanisms leading to their transcriptional silencing are yet to be defined. Earlier studies have shown that methylation at *dCK* gene is responsible for its silencing [19] however; this remains to be demonstrated for the *hENT1*.

Hydralazine is a small-molecule DNA demethylating agent [20] known to demethylate gene promoters and to induce gene reactivation in vitro [21]–[24] and in vivo [25]. Used in combination with the histone deacetylase inhibitor valproic acid reactivates the expression of genes in cancer patients [26]–[28]. In addition, hydralazine has been shown to reverse doxorubicin resistance in a model of breast cancer [29]. As most of the work on gemcitabine resistance has been done in pancreatic cancer cell lines and, this drug has been extensively evaluated in cervical cancer [30] we wanted to investigate whether epigenetic mechanisms are responsible for acquiring resistance to this agent and if hydralazine could restore gemcitabine sensitivity in cervical cancer cells. Our results demonstrate that in this model, hydralazine reverses gemcitabine resistance in a DNA methylation-independent manner.

## Results

**Figure 1:**
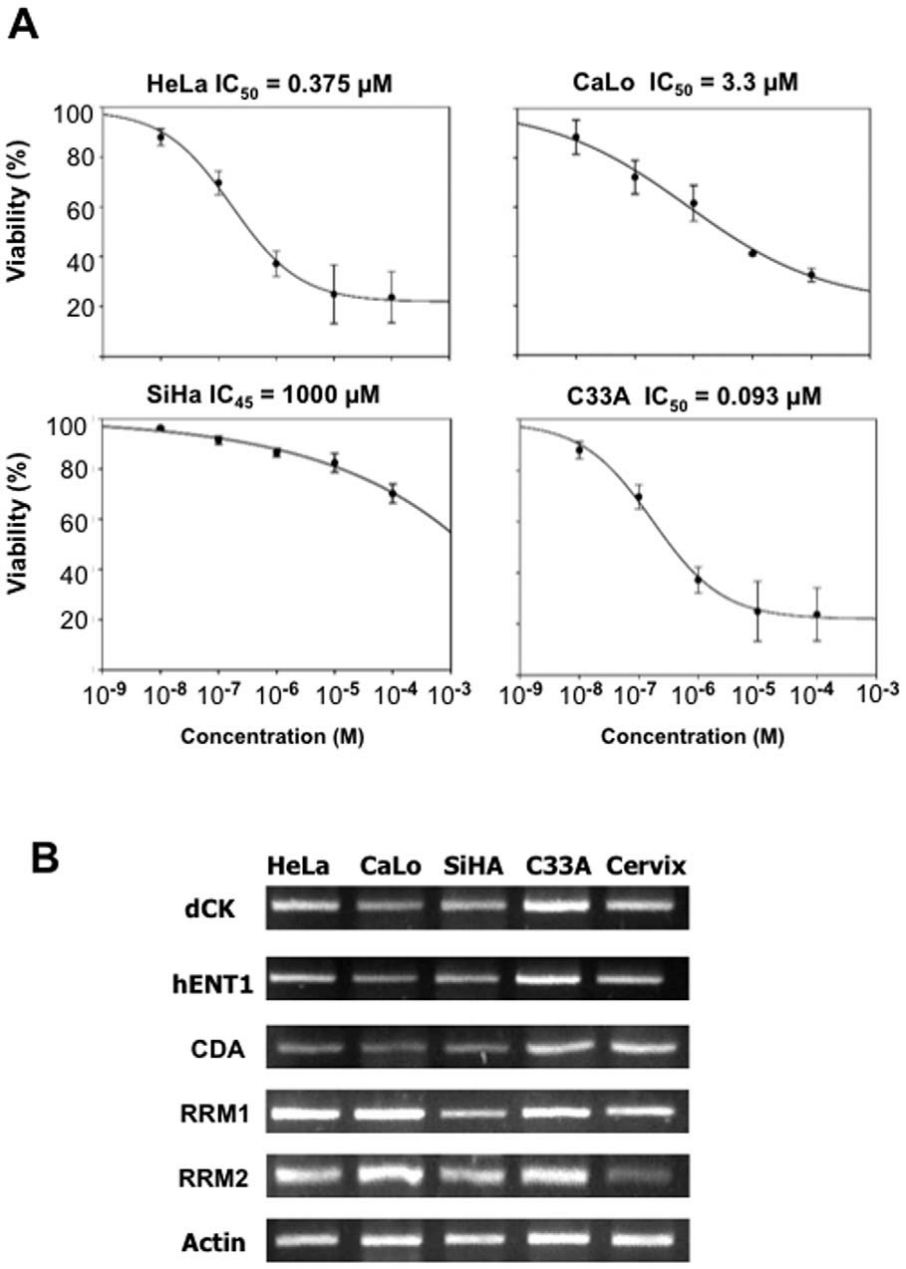
Gemcitabine sensitivity in cervical cancer cells and basal gene expression. A. The IC_50_ of gemcitabine for HeLa, CaLo, SiHa and C33A cells were 3.3 µM, 0.3 µM, >1000 µM and 0.1 µM respectively as evaluated with the crystal violet assay. B. Basal expression of *hENT1*, *dCK*, *RRM1*, *RRM2*, *CDA* genes as evaluated by RT-PCR. There was no correlation between IC_50_ and the intrinsic sensitivity/resistance status. Expression was adjusted to the expression in normal cervix.

Cervical cancer cell lines were examined for the basal expression of *hENT1*, *dCK*, *RRM1*, *RRM2*, *CDA* genes and then their sensitivity to gemcitabine was evaluated. The IC_50_ in SiHa cell line was >1000 µM, and it was arbitrarily considered as a primary resistant. The IC_50_ for the other cell lines were: 3.3 µM, 0.3 µM and 0.1 µM for CaLo, HeLa and C33A cells, respectively (Figure 1A). As shown in figure 1B, the basal expression of genes coding for gemcitabine transport and metabolism which were adjusted to actin and in reference to normal cervix, varied among the cell lines and a relationship between levels of these genes with the intrinsic sensitivity/resistance status to gemcitabine in these cell lines was not found.

**Figure 2:**
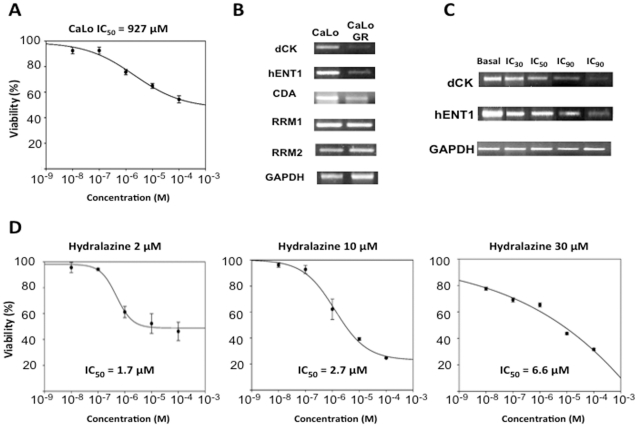
Gemcitabine sensitivity, gene expression and effects of hydralazine in CaLo cells. A. After acquiring gemcitabine resistance, the IC_50_ in CaLoGR cells was 927 µM (280-fold concentration). B. Expression of *hENT1*, *dCK* and *CDA* were reduced almost by half, RRM1 and RRM2 had minor changes. C. The reduction of *hENT1* and *dCK* genes occurred progressively as resistance developed. D. Treatment of CaLoGR cells with hydralazine at 2 µM for 10 days, 10 µM and 30 µM for 5 days reverted gemcitabine resistance. The corresponding IC_50_s for these treatments were 1.7 µM, 2.7 µM and 6.6 µM respectively.

To investigate whether the induction of gemcitabine resistance is related to changes in the expression of *hENT1*, *dCK*, *RRM1*, *RRM2*, *CDA* genes, CaLo cells were exposed to increasing concentrations of gemcitabine and once cells became resistant they were treated with hydralazine at different times and concentrations. Figure 2A shows that after five weeks, CaLo cells acquired resistance to this antimetabolite and were able to grow at 927 µM of gemcitabine (280-fold concentration). The resistance state was accompanied by downregulation by half of *hENT1* and *dCK*. *RRM1* and *RRM2* genes had minor changes whereas *CDA* was also reduced (Figure 2B). In addition, figure 2C shows that the down regulation of *hENT1* and *dCK* genes occurred progressively as resistance developed. Treatment of CaLoGR cells with hydralazine at 2 µM for 10 days, 10 µM and 30 µM for 5 days was able to revert gemcitabine resistance as shown in figure 2D. The corresponding IC_50_s for these treatments were 1.7 µM, 2.7 µM and 6.6 µM respectively.

**Figure 3:**
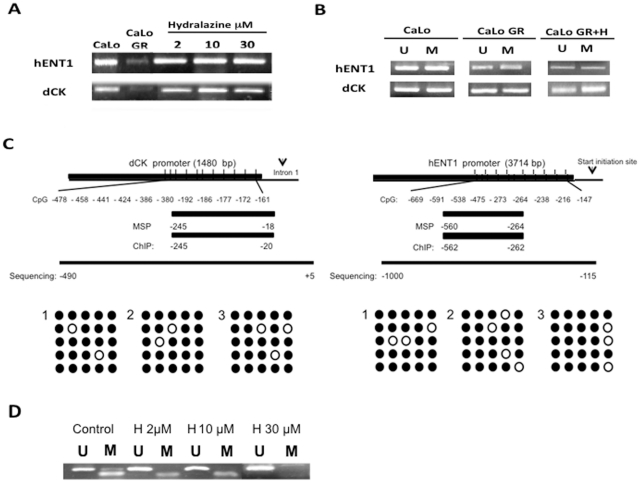
Effect of hydralazine upon *hENT1*, *dCK* expression and promoter methylation. A. Hydralazine at the three conditions tested (2 µM for 10 days, 10 µM and 30 µM for 5 days) restored the expression of these genes. B. *hENT1*, *dCK* genes were partly methylated basally and promoter methylation did not change in the resistant cells neither after hydralazine treatment as evaluated by MSP. C. Maps of the promoters at these genes showing the CpG islands density and distribution as well as the positions of primers for MSP, ChIP and sequencing. It is also shown by sequencing that the CpG methylation did not change in *dCK* and *hENT1* genes neither in the resistant cells neither when treated with hydralazine. Empty and filled circles represent demethylated and methylated CpGs in five independent sequencences. D. Hydralazine was able to demethylate the *DAPK* promoter in the CaLo cell line.

To determine whether the reversion of the resistance by hydralazine which is a weak DNA demethylating agent is accompanied by changes in the expression of *hENT1* and *dCK* genes, a RT-PCR of these two genes was performed. The results in figure 3A shows that as expected hydralazine led to the re-expression of these genes in the three treatment conditions evaluated. DNA promoter hypermethylation has been shown to silence genes in chemotherapy resistance models therefore the methylation status at these promoters was evaluated by MSP. Interestingly, *hENT1* and *dCK* promoters were partially methylated even in the basal state and showed no hypermethylation in the resistant CaLoGR cell line. Hydralazine at 2 µM, 10 µM or 30 µM failed to demethylate these promoters as shown by MSP in figure 3B. To confirm this finding, six independent clones (basal, resistant and resistant treated with hydralazine) were bisulfite sequenced but no demethylation was observed in the CpG evaluated (Fig. 3C). The gene reactivation therefore was independent of its demethylating effect, suggesting that other epigenetic mechanisms could account to down regulate and reactivate these genes in this model. To confirm that the lack of demethylating effect of hydralazine upon *dCK* and *hENT1* was not due to its weak demethylating ability, figure 3D shows that in the same cell line and conditions, the gene *DAPK* was demethylated with hydralazine at 2 µM 10 µM and 30 µM.

**Figure 4:**
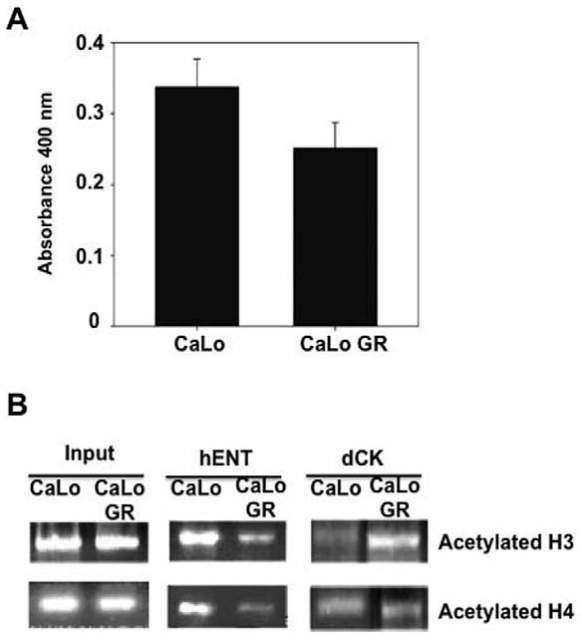
HDAC activity and promoter acetylation in CaLo cells. A. Total histone deacetylase activity showed no major changes between basal and resistant cells, actually a small decrease was observed in the resistant cells as evaluated with a HDAC activity kit. B. Total acetylation of H3 and H4 as evaluated by ChIP showed a decrease in H3 and H4 acetylation at the *hENT1* promoter, a mild increase in acetylation of H3 and not change in H4 at *dCK* promoter.

An increase of histone deacetylase activity has been shown to induce gene silencing in some models. To determine whether this phenomenon participates in gemcitabine resistance in cervical cancer, the deacetylase activity was measured in CaLo cells using a kit which contains the prototype HDAC inhibitor trichostatin A as a positive control. Figure 4A shows that no changes in HDAC activity were observed; actually a small decrease in deacetylase activity was observed in the resistant cells. The evaluation of the total acetylation at H3 and H4 in *hENT1* and *dCK* gene promoters by ChIP assays while showed a decrease in H3 and H4 acetylation at the *hENT1* promoter, a mild increase in acetylation of H3 was observed in the *dCK* promoter and no change for H4 acetylation (Figure 4B). These apparently opposite results argue against a major role of H3 and H4 acetylation to explain the silencing of these genes by this histone modification. Further, valproic acid treatment at 1 mM for 5 days failed to reactivate the expression of these genes and to reverse gemcitabine resistance (not shown).

**Figure 5:**
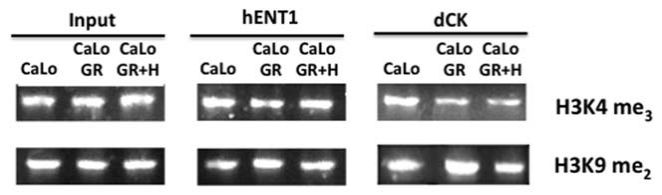
H3K9 and H3K4 methylation. For *hENT1* gene, no changes were observed in H3K4m3 methylation but H3K9me2 mildly increased in the resistant cells and decreased after hydralazine treatment. In *dCK* gene there was a slight reduction in H3K4me3 in the resistant cells which was not modified by hydralazine. The methylation at H3K9me2 mildly increased in resistant cells and reduced after hydralazine. The changes in band intensity were normalized against their respective inputs.

**Figure 6:**
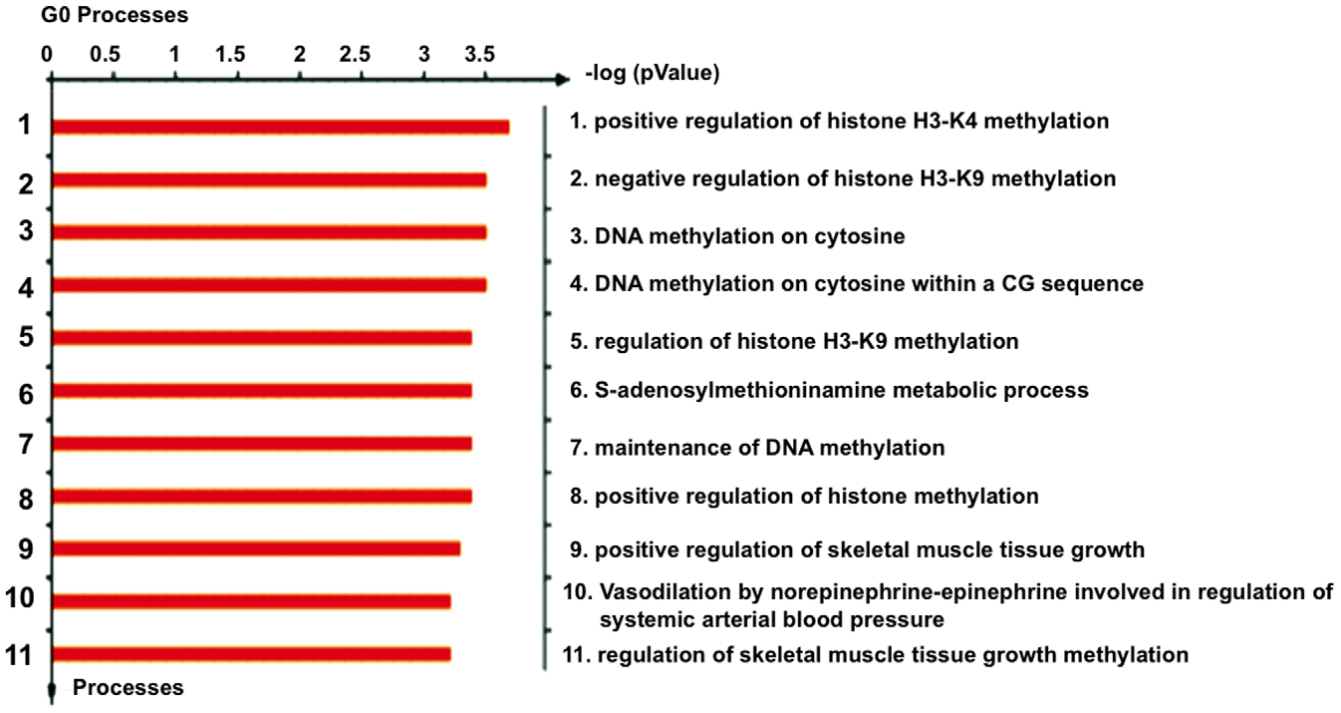
GO processes affected by hydralazine. MetaDrug™ program predicted that hydralazine may affect not only DNA methylation in cytosine but also can be involved in negative regulation of H3K9 methylation.

**Figure 7:**
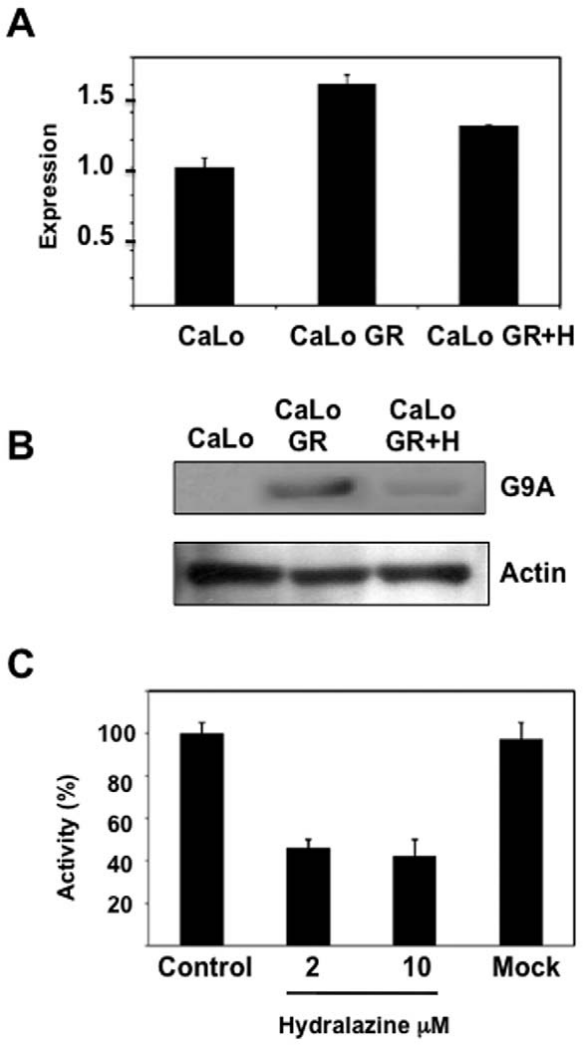
Effect of hydralazine upon G9A histone methyltransferase. A. Quantitative RT-PCR of *G9A* shows that resistant CaLoGR cells had an increase as compared to basal and that hydralazine reduces its expression (basal vs resistant, p<0.05). B. At protein level, there was an increase in G9A in the resistant cells that decreased after hydralazine treatment. C. H3K9 methyltransferase *in vitro* inhibition assay. Hydralazine at 2 µM and 10 µM strongly reduced H3K9 methylation (untreated vs hydralazine, p<0.05).

**Figure 8:**
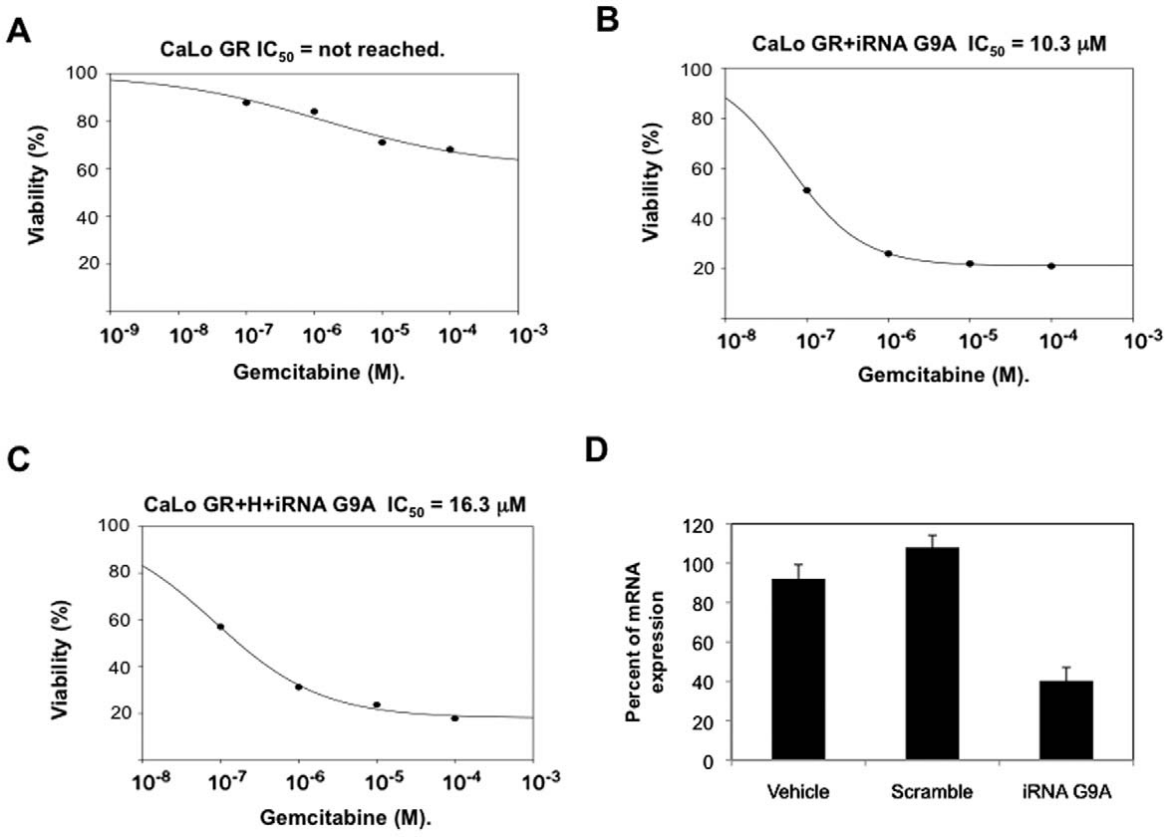
iRNA assay for G9A and gemcitabine resistance. A. Inhibitory concentration of gemcitabine in CaLo cells not reached. B. Depletion of G9A restored gemcitabine sensitivity (IC_50_ 10.3 µM). C. No further changes in IC_50_ were seen when hydralazine was added to the G9A depleted cells. D. qPCR of G9A confirming the partial depletion of *G9A* messenger.

To gain further insight into the epigenetic mechanisms of *hENT1* and *dCK* silencing in this model of gemcitabine resistance, histone methylation was assayed. Methylation of H3K9 is a known repressive mark while methylation at H3K4 is activating, hence the methylation at these lysines was evaluated by ChIP analysis in the basal state, in resistant cells and after hydralazine treatment. Figure 5 shows that for the *hENT1* gene, no changes were observed in H3K4me3 methylation but H3K9me2 mildly increased in the resistant cells and decreased after hydralazine treatment. Regarding the *dCK* gene a slight reduction in H3K4me3 was observed in the resistant cells which was not modified by hydralazine. Nevertheless, the methylation at H3K9me2 mildly increased in resistant cells and reduced after hydralazine. The changes in band intensity were normalized against their respective input intensity. As the DNA demethylating agent 5-aza-CdR has shown to decrease H3K9me2, we used the MetaDrug™ program to uncover whether hydralazine may be involved in the regulation of H3K9 methylation. As shown in Figure 6, hydralazine may affect not only DNA methylation in cytosine but also can be involved in negative regulation of H3K9 methylation. To confirm these predictions the mRNA expression of *G9A* was evaluated in CaLo cells by qPCR. The results show that the expression of this gene increased in the resistant cells whereas hydralazine decreased its level (figure 7A). Similar results were obtained by western blot, G9A protein increased in the resistant cells and decreased after hydralazine treatment (Figure 7B). To further investigate the G9A inhibitory ability of hydralazine, a H3K9 methyltransferase *in vitro* inhibition assay was performed using the *EpiQuik*™ Histone Methyltransferase Activity/Inhibition Assay Kit (H3-K9). Hydralazine at 2 µM and 10 µM strongly reduced H3K9 methylation (figure 7C). To confirm the biological significance of this in vitro finding, knockout of the G9A gene by means of an iRNA assay showed that transfected CaLo cells regained sensitivity to hydralazine which was not further modified when these cells also were treated with hydralazine (Fig. 8).

## Discussion

The results of this study of gemcitabine resistance in cervical cancer cells and its reversion with the weak DNA demethylating agent hydralazine show that the development of resistance is accompanied by down-regulation of key genes for gemcitabine's intracellular uptake and metabolism, *hENT1* and *dCK*. Interestingly, this down-regulation was not due to gene promoter hypermethylation as demonstrated by MSP and bisulfite sequencing; nevertheless, hydralazine was able to reactivate their expression and to revert gemcitabine resistance. These data suggest that other epigenetic mechanisms operate to silence chemotherapy resistance genes and that hydralazine has DNA methylation-independent effects, most likely through affecting negatively the regulation of H3K9 methylation as predicted by the METADRUG™ program.

These findings are important to gain further knowledge onto the mechanisms of chemotherapy drug resistance. Gene inactivation by DNA methylation was the first epigenetic mechanism shown to be directly involved in the acquisition of chemotherapy resistance in *in vitro* models [31], [32] however, as the knowledge on epigenetic processes has evolved, the participation of other epigenetic players such as histone deacetylases both zinc-and NAD-dependent as well as histone methyltransferases, histone demethylases and microRNAs in the inactivation process of genes involved in chemotherapy sensitivity and resistance has been uncovered [33]–[38]. Our results strongly suggest that in this model of gemcitabine resistance in cervical cancer, methylation at H3K9 could be the main mechanism for silencing the expression of *hENT1* and *dCK* genes which paves the way for further testing the participation of this histone modification in other models of chemotherapy resistance. The relevance of this finding increases as inhibitors of G9A histone methyltransferases are being available for study [39].

Early studies have shown that the DNA methylation inhibitors induced *dCK* re-expression in the CEM/dCK- cells [19] and that the lack of expression of this key gene for activation of several nucleoside analogs including gemcitabine occurs by promoter methylation [40]–[43]. So far no other epigenetic mechanism for silencing this gene has been described in chemotherapy resistance models. Likewise, lack of expression or low levels of hENT1 strongly correlate with resistance to gemcitabine and other nucleoside analogs [6], [9], [10], [44]. Though several membrane transporter coding-genes such as hOAT3 (SLC22A8), the solute carrier family 5 iodidetransporter (SLC5A8), the reduced folate carrier (RFC), the cellular retinol-binding protein 1, and the human Na+/I-symporter are known to be down regulated by DNA methylation and reactivated by DNA methyltransferase inhibitors [45]–[49], here we were not able to demonstrate that promoter methylation at *hENT1* accounts for its observed downregulation, yet its reactivation was achieved by hydralazine through a DNA methylation-independent mechanism suggesting that indeed *hENT1* is down regulated by an epigenetic effect most likely through G9A histone methyltransferase-mediated H3K9 methylation.

Despite that both genes contain CpG islands at their promoters [50], [51] and that at least for the *dCK* gene it has consistently been shown that its promoter is methylated in resistant cells [19], in our model there is no correlation between the promoter methylation at these promoters and expression. Nevertheless, the demethylating effect upon other genes (*DAPK*) was proven, ruling out that hydralazine has no demethylating effect. These data led us to search for other epigenetic mechanisms that could account for gene silencing. No consistent changes in histone deacetylase nor in histone acetylation at these gene promoters were observed ruling out this histone modification as a silencing mechanism for these genes in this model. As the DNA methyltransferase inhibitor 5-aza-CdR decitabine may exhibit alternative mechanisms of action to DNA methylation with regard to transcriptional reactivation such as histone H3K9 demethylation in the regulatory regions of silenced genes [52] we performed a search in the METADRUG™ program to get clues onto other possible effects of hydralazine. Our results showed that indeed hydralazine is predicted to be involved in the negative regulation of H3K9 methylation. This result led us to analyze by ChIP analysis for changes in the H3K9me2 at both *dCK* and *hENT1* promoters in the CaLoGR cells. Both promoters contained H3K9me2 in untreated cells, slightly increased in the resistant state, and this silencing mark was partially relieved by hydralazine. We must however, stress that these changes though in H3K9m2 though consistent in three separate assays, were mild which can be explained on the basis that at least eight histone methyltransferases including G9A also have H3K9 as their methylation target [52].

To further confirm the finding, a quantitative RT-PCR showed that the expression of G9A was increased in the resistant cells as compared to sensitive cells and levels did decrease after treatment with hydralazine. Nevertheless, a dramatic reduction at the protein level of this histone methyltransferase was observed by western blot in the resistant cells after hydralazine treatment. Remarkable, these results are very similar to those found with 5-aza-CdR in a breast cancer model of MASPIN re-expression where reductions in G9A histone methyltransferase protein levels are not due to effects on G9A gene expression [53]. Additional support for this effect of hydralazine was obtained by an in vitro assay of H3K9 methylation showing that hydralazine inhibits the activity of this histone methyltransferase. The biological meaning of these observations was proven by showing that depleting G9A in the resistant CaLo cells led to regain sensitivity to gemcitabine by these cells and that higher sensitivity to gemcitabine was not achieved by adding hydralazine to the G9A depleted cells arguing against an *off-target* effect of hydralazine.

Of note, the METADRUG™ program also indicated that hydralazine could act positively in the regulation of H3K4 methylation which was not observed in our study, however, recent data from our group indicate that in several cancer cell lines treatment with hydralazine and valproic acid led to over-expression of MIC A and MIC B ligands which was accompanied by an increase in H3K4 methylation at these gene promoters suggesting that hydralazine could have this effect upon this histone mark [54].

Either intrinsic or acquired drug resistance is a complex and pleiotropic phenomenon and other potential players participating in gemcitabine resistance in this model were not investigated in this study. For instances, despite *RRM1* and *RRM2* over expression has been related to drug resistance [55] no major changes were observed at these genes in this study. Paradoxically, CD gene was down regulated in the resistant cells despite its over-expression has consistently been shown related with resistance to gemcitabine and other nucleoside analogs [56]. This finding further underscores the complexity of drug resistance which seems to be gene and cell model-specific.

The findings of this study are of potential clinical relevance at least for this model of drug resistance. Gemcitabine is frequently used for cervical cancer either concurrent to radiation or in combination with cisplatin for advanced stages [3]. Acquired resistance to this drug may be responsible for treatment failure; hence, hydralazine may prevent resistance and potentially increase the efficacy of gemcitabine. In addition, the uncovering that H3K9 methylation may lead to chemotherapy resistance deserves its testing in other experimental models.

## Materials and Methods

### Cell culture

The cervical cancer cell lines HeLa, SiHa and C33A were obtained from the ATCC. The CaLo cell line was a kindly donated by Dr. Monroy-Garcia [57]. Cell lines were cultured at 37°C in humidified atmosphere containing 5% C0_2_ in DMEM supplemented with 10% fetal calf serum (Gibco, Grand Island, NY).

### Cytotoxicity assays

Cells were seeded into 6-well microtiter Falcon plates (Becton Dickinson, Franklin Lakes, NJ) at 2×10^3^ cells/well into 0.2 ml of complete medium and then treated for 24 hours with gemcitabine at concentrations ranging from 1×10^−8^ M to 1×10^−4^ M. Thereafter, the medium containing gemcitabine was removed and fresh medium was added. After 72 h medium was aspirated and replaced for 10 minutes with 50 µL of 0.75% crystal violet in 50% ethanol, 0.25% NaCl, and 1.75% formaldehyde solution. Cells were then washed with water, air-dried, and the dye eluted with PBS+1% sodium duodecyl sulfate (SDS) solution. Cell viability was assessed by dye absorbance measured at 570 nm on an automated ELISA reader. All assays were performed in triplicate. The cytotoxic effect of each treatment was expressed as a percentage of cell viability relative to untreated control cells (percentage of control) and is defined as [A_570 nm_ treated cells/A_570 nm_ non-treated cells]×100.

### Induction of gemcitabine resistance and hydralazine treatment

CaLo cell line was cultured at 37°C in humidified atmosphere containing 5% C0_2_ in DMEM supplemented with 10% fetal calf serum (Gibco, Grand Island, NY). Increasing doses of gemcitabine (IC_30_, IC_50_, IC_80_ and finally two steps at IC_90_ were added weekly to induce gemcitabine resistance. After adding IC_90_ twice, the cytotoxic assay was repeated to confirm gemcitabine resistance.

Gemcitabine-induced resistant CaLo cells (CaLoGR), were cultured at 37°C in humidified atmosphere containing 5% C0_2_ in DMEM supplemented with 10% fetal calf serum (Gibco, Grand Island, NY). Thereafter, hydralazine was added at 10 µM and 30 µM for 5 days and 2 µM for 10 days. The medium containing the drug was replenished daily.

### RT-PCR

RNA was isolated from cell lines by standard methods. Thereafter 5 µg of RNA were treated with DNAse. Reverse transcription was done using the RNA kit PCR Core Gene Amp^R^ (Applied Biosystems Roche) following the provider recommendations. *GAPDH*, *hENT1*, *dCK*, *RRM1*, *RRM2*, *CDA* genes were amplified using the following oligonucleotide primers: *GAPDH*:S: 5′-GAAGGTCGGAGTCAACGGATTT-3′, AS:5′-ATGGGTGGAATCATATTGGAAC-3′ *hENT1*: S: 5′GCAAAGGAGAGGAGCCAAGA-3′, AS: 5′CCCAACCAGTCAAAGATATTG-3′ *dCK*: S: 5′-CGATCTGTGTATAGTGACAG-3′, AS: 5′GTTGGTTTTCAGTGTCCTATG-3′, *RRM1*: S: 5′-GCAGCTGAGAGAGGTGCTTT-3′, AS: 5′-CAGGATCCACACATCAGACA-3′, RRM2: S: 5′-GAGTTCCTCACTGAGGCC-3′, AS: 5′-TTAGAAGTCAGCATCCAAG-3′, *CDA*: S:5′ GTTGCCTTGTTCCCT TGTAA-3′, AS: 5′-TCTTGCTGCACTTCGGTATG-3′. PCR was performed in a total volume of 25 µl containing cDNA, 20 pmol of primers, 200 µM dNTPs, 0.25 U Taq polymerase, and 1× buffer supplied by the manufacturer (Applied Biosystems, Foster City, CA). PCRs were initiated by a denaturation step at 94°C for 5 min, followed by 30 cycles at 94°C for 35 sec, 59°C for 35 sec, and 72°C for 45 sec; a final extension was performed at 72°C for 7 min. Products were electrophoresed in 2% agarose gels. *G9A* gene expression was evaluated by quantitative RT-PCR (iCycler iQ, Bio-Rad, Hercules, CA), using SYBR Green I dye (Bio-Rad, Hercules, CA), using the following primers: *G9A*; Sense 5′-CTCCGCTGATTTTCGAGTGTAA-3′ and the antisense 5′-GTCGAAGAGGTAAGAATCATCC-3′. *GUSB* (human beta glucuronidase) specific primers were used as endogenous control; sense 5′-CCTGTGACCTTCTGAGCAA-3′ and antisense 5′-AAACCCTGCAATGGTTTCTG-3′. The PCR started by incubation at 95°C for 1 min, followed by PCR cycles of 35 sec, at 94°C, 35 sec, at 59°C, 50 sec, at 72°C, with a final extension at 72°C for 7 min. The number of PCR cycles was determined experimentally. Data were analyzed using the 2−ΔΔCT method and reported as the fold change in gene expression normalized to the endogenous control gene (*GUSB*) and relative to cells without treatment.

### iRNA transfection assay

Gemcitabine acquired resistant cells were seeded into 24 microtitier Falcon (Becton Dickinson, Franklin Lakes, NJ) at 1.5×10^5^ cells/well into 0.2 ml of optimem and after 24 hours were transfected with lipofectamine and *G9A* siRNA (Ambrion cat #439242). Negative control was done with lipofectamine RANiMAX containing siRNA Scramble (Ambrion, cat # 4390844). Cells were cultured were cultured at 37°C in humidified atmosphere containing 5% C0_2_. After 72 h medium was aspirated and RNA was extracted, as well as a cytotoxicity assay was done.

### Western blot analysis

Whole cell extracts were prepared in lysis buffer containing 50 mM tris-HCl pH 7.4, 150 mM NaCl, 0.5% Nonidet P-40, 1 mM EDTA, 1 mM phenylmethylsulfonyl fluoride (PMSF), 0.2 mM Na_3_VO_4_ and a proteases inhibitor cocktail (Sigma). Extracted proteins were analyzed by sodium duodecyl sulfate-polyacrylamide gel electrophoresis (SDS-PAGE)/immunobloting with antibodies recognizing G9A protein (Rabbit polyclonal IgG, anti G9A protein (Millipore, Billerica, MA), and B-Actin protein (Santa cruz Biotechnology). Protein samples were separated in 10% polyacrylamide gels. Gels were transferred into nitrocellulose membranes. Species-specific immunoglobulin G-horseradish peroxidase (IgG-HRP) secondary antibodies were purchased from Santa Cruz Biotechnology. Membranes were revealed with a chemoluminiscent substrate (Millipore).

### Histone deacetylase assay

Assays were performed using the colorimetric HDAC activity assay from BioVision (BioVision Research Products, Mountain View, CA) according to manufacturer instructions. Briefly, 50 µg of nuclear extracts from CaLo cell line were diluted in 85 µL of ddH_2_0; then, 10 µL of 10× HDAC assay buffer were added followed by addition of 5 µL of the colorimetric substrate; samples were incubated at 37°C for 1 h. Subsequently, the reaction was stopped by adding 10 µL of lysine developer and left for additional 30 min at 37°C. Samples were then read in an ELISA plate reader at 400 nm. HDAC activity was expressed as relative OD values per µg of protein sample. The kit contains negative and positive controls that consist of nuclear extract of HeLa treated or not with trichostatin A, respectively.

### Methylation-Specific PCR (MSP) and Bisulfphite sequencing-PCR (BSP)

Genomic DNA was extracted with the extraction kit Wizard Genomic DNA purification kit (Promega, Madison, WI), according to manufacturer instructions. DNAs were quantified spectrophotometrically and stored at −20°C. Bisulfite modification was done with the Methylation Direct™ KIT (Zymo Research, Irvine CA) according with the manufacturer instructions. MSP primers were designed with the Methprimer program [58] to analyze the methylation status within the promoter region of *hENT1* and *dCK* genes. *dCK U*, S: 5′- TGGGGTAGAGGTTTTTTGTTATATG- 3′, AS: 5′- AACTAAAAACACTAACAAACCTACAAA- 3′, *dCK* M, S: 5′- TGGGGTAGAGGTTTTTCGTTATAC-3′, AS: 5′-CAACTAAAAACACTAACGAACCTACG- 3′. *HENT1* U, S: 5′- TTTTGTTTATTAGGAGAGAGTAGTTGT-3′, AS: 5′-ATTAAAAAATCTAAAAACCACCAAA-3′. *HENT1* M, S: 5′- GGTTTTGTTTATTAGGAGAGAGTAGTC-3′, AS: 5′-ATTAAAAAATCTAAAAACCACCGAA-3′, PCR was performed in a total volume of 25 µl containing bisulfite-modified DNA, 20 pmol of primers, 200 µM dNTPs, 0.25 U Taq polymerase, and 1× buffer supplied by the manufacturer (Applied Biosystems, Foster City, CA). PCRs were initiated by a denaturation step at 94°C for 5 min, followed by 50 cycles at 94°C for 35 sec, 57°C for 35 sec, and 72°C for 45 sec; a final extension was performed at 72°C for 7 min. *DAPK* promoter methylation was evaluated by MSP in CaLo cell line, before and after hydralazine treatment with the following primers: U, S: S: 5′-GGAGGATAGTTGGATTGAGTTAAT-3′; AS:5′- CAAATCCCTCCCAAACACCAA-3′; M, S:5′-GGATAGTCGGATCGAGTTAACGT-3′; AS: 5′- CCCTCCCAAACGCCGA-3′. The PCR mixture contained 2 ml of 10× PCR buffer, 0.5 U of Taq Gold polymerase, dNTP's (each 1.25 mM), 100 ng of primers and bisulfite-modified DNA in a final volume of 20 µl. Conditions were 95°C for 10 min followed by 35 cycles of 95°C for 30 sec, 56°C for 30 sec and 72°C for 35 sec, with a final extension cycle of 72°C for 6 min. Primers for BSP were also designed using methprimer program [58], to sequence from −1000 to −115 region of *hENT1* promoter: *hENT1*, *S*: 5′-GGTGGAGAGATTAGATTTGTAGAG-3′, *AS*: 5′-AATCAAAAAAAACAAACAAAAAAAC-3′; and *hENT1*, *S*:5-TTATATAAATGGGGAGTAGGAGAGG-3′, *AS*: 5′-CCCAAAAACTTCCTAATTACTAACC-3′; and from −420 to +5 region of *dCK* promoter: *dCK,S*: 5′-TTTGTTTATTTTTAATAGGTTTATTAGAGA-3′, *AS*: 5′-TCTACCCCAAACCAACAAAC-3′; and *dCK*, *S*: 5′-GGTTTGGGGTAGAGGTTTTT-3′, AS: 5′-CTAAACCAAATCCTAACCTACC-3′. PCR was performed in a total volume of 25 µL containing cDNA, 20 pmol of primers, 200 µM dNTPs, 0.25 U Taq polymerase, and 1× buffer supplied by the manufacturer (Applied Biosystems, Foster City,CA). PCRs were initiated by a denaturation step at 94°C for 5 min, followed by 50 cycles at 94°C for 35 sec, 57°C for 35 sec, and 72°C for 45 sec; a final extension was performed at 72°C for 7 min. PCR amplicons were purified and cloned into the pGEM-T-Easy vector (Promega) for sequencing using the T7 primer. At least five clones were selected randomly for DNA sequencing. Sequencing reactions were electrophoresed on an ABI3100 genetic analyzer. Electropherograms were analyzed in both sense and antisense direction for presence of methylated or unmethylated CpG islands.

### ChIP analysis

Cells were treated with 1% formaldehyde at room temperature for 10 min under constant agitation. The reaction was stopped by the addition of glycine to a final concentration of 125 mM. Cells were washed twice in ice-cold PBS 1×, resuspended in lysis buffer (50 mM Hepes-KOH pH7.9, 10 mM EDTA pH 8.0, 1% SDS) containing protease inhibitors, and sonicated on ice until crosslinked chromatin was sheared to an average DNA fragment length of 0.5–1 Kb. After centrifugation, soluble cross linked chromatin was diluted 1∶10 in immunoprecipitation (IP) buffer (10 mM Hepes-KOH pH 7.9, 1% Triton X-100, 150 mM NaCl, and protease inhibitors) divided into aliquots and stored at −70°C. Protein A-Agarose (Millipore) was blocked with BSA (1 mg/mL) and Salmon-sperm DNA (Sigma-Aldrich) and in IP buffer for 4–6 h at 4°C and subsequently washed extensively with IP buffer before use. Chromatin preparations were pre-cleared by incubation with blocked protein A-agarose for 2 h at 4°C. The protein A-agarose was removed by centrifugation; the pre-cleared chromatin was incubated with antibody (anti acetyl-histone H3, anti-acetyl-histone H4, anti-H3K4me3, and anti-H3K9me2 (Millipore, Billerica, MA) for 12–14 h at 4°C. Immunoprecipitates were recovered by incubation with fresh blocked protein A-agarose for 2 h at 4°C, followed by low-speed centrifugation. The pellets were washed three or four times with IP buffer, three times in wash buffer (10 mM Tris-HCl pH 8.0, 0.25 mM LiCl, 0.5% NP-40, 0.5% sodium deoxycholate, 1 mM EDTA pH 8.0), and three times in Tris-EDTA (TE) pH 8.0. Precipitates were then extracted by incubation with elution buffer (50 mM Tris pH 8.0, 1% SDS, 50 mM NaHCO_3_, 1 mM EDTA pH 8.0), and formaldehyde crosslinks were reversed by treatment with a 1/25 volume of 5 M NaCl for 8 h at 65°C. The DNA was purified by extraction with phenol and ethanol precipitation and analyzed by PCR with the specific primers (for *hENT1*, *S*: 5′-CCTGTGACAGAGAGGAACTAAG-3′, *AS*: 5′-GCGACAACATCGATGATGACTG-3′; for *dCK*, *S*: 5′-CCTCCCCACCCGACTCCGGAACC-3′, AS: 5′-CAGCTGAGGACACTGGCGGGCCTG-3′). PCR was performed in a total volume of 25 µl containing DNA, 20 pmol of primers, 200 µM dNTPs, 0.25 U Taq polymerase, and 1× buffer supplied by the manufacturer (Applied Biosystems, Foster City, CA). PCRs were initiated by a denaturation step at 94°C for 5 min, followed by 35 cycles at 94°C for 35 sec, 59°C for 35 sec, and 72°C for 45 sec; a final extension was performed at 72°C for 7 min. PCR products were separated on a 2% agarose gel and visualized by ethidium bromide staining.

### Prediction of processes affected by hydralazine

Hydralazine structure (primary accession number DB01275) was downloaded from (www.drugbank.ca/drugs/DB01275) and loaded into the MetaDrug™ (http://www.genego.com) program. MetaDrug™ combines a suite of chemical structural analysis tools (metabolite prediction, QSAR, structural similarity searching), a comprehensive structure-activity database, and a systems biology database of molecular interactions (protein-protein, compound-protein, protein-enzymatic reaction, compound-enzymatic reaction), canonical signaling and metabolic pathways, and gene-biological property associations (gene-function, gene-disease, gene-toxicity, etc.). MetaDrug™ was set up to compile list of targets based on known targets of the hydralazine and targets of compounds with at least 70% similarity to input molecule. The resulting list was subjected to enrichment analysis across GeneGo Pathway Maps.

### In vitro Histone methyltransferase assay

H3K9 methyltransferase *in vitro* inhibition by hydralazine was evaluated using the *EpiQuik*™ Histone Methyltransferase Activity/Inhibition Assay Kit (H3-K9) Epigentek, Brooklyn, NY) which contains recombinant G9A as the control enzyme. The assay was done following to the manufaturer's protocol.
